# Insights into Clustering Patterns in Romania’s 2020–2024 Measles Cases

**DOI:** 10.3390/epidemiologia7010011

**Published:** 2026-01-07

**Authors:** Valerian-Ionuț Stoian, Cătălin Pleșea-Condratovici, Mădălina Nicoleta Matei, Iulia Draghiev, Liliana Baroiu, Carmina Mușat, Mihaela Patriciu, Valerii Luțenco, Mariana Daniela Ignat, Mihaela Debita

**Affiliations:** 1 Medical Department, Faculty of Medicine & Pharmacy, “Dunărea de Jos” University of Galați, 800008 Galați, Romania; valerian.stoian@ugal.ro (V.-I.S.); iuliadraghiev@yahoo.com (I.D.); patriciu_mihaela@yahoo.com (M.P.); mdebita@ugal.ro (M.D.); 2 National Institute for Public Health, 050463 Bucharest, Romania; 3 Department of Morphological and Functional Sciences, Faculty of Medicine and Pharmacy, “Dunarea de Jos” University, 800008 Galati, Romania; carmina.musat@ugal.ro; 4 Department of Dental Medicine, Faculty of Medicine and Pharmacy, “Dunarea de Jos” University, 800008 Galati, Romania; 5 Department of Clinical Medicine, Faculty of Medicine and Pharmacy, “Dunarea de Jos” University, 800008 Galati, Romania; lilibaroiu@yahoo.com (L.B.); mariana_daniela52@yahoo.com (M.D.I.); 6 Department of Clinical Surgery, Faculty of Medicine and Pharmacy, “Dunarea de Jos” University, 800008 Galati, Romania; valerii.lutenco@ugal.ro

**Keywords:** measles, outbreak, Romania, basic reproduction number

## Abstract

**Background and objectives**: During an outbreak, measles cases tend to aggregate into increasingly bigger clusters that show specific characteristics, different from the non-cluster cases. As the measles threat continues throughout Europe in 2025 with a high notification rate in Romania as well, exploring how clustering affects the disease propagation can provide additional insights into how to improve measles surveillance and control. **Methods**: National measles cases from 2020 to 2024 have been split into cluster (at least three related cases) and non-cluster-related cases and analyzed comparatively based on vaccination status, disease-related data (hospitalization) and patient-related data (age, location). Large outbreaks with at least 150 cases, allowing for more comprehensive R_0_ analysis, have been described and the basic reproduction numbers computed for each of them. **Results**: There are statistically significant differences in vaccination status, age, and hospital stay between outbreak and non-outbreak cases. Large outbreaks (≥150 cases) show a high degree of variability, with R_0_ values varying from as low to 1 to as high as 3.92, indicating limited measles transmission control. **Conclusions**: The findings in this research highlight the critical impact of clustering on measles transmission dynamics during outbreaks. Significant differences in vaccination status, age, and hospitalization rates between cluster and non-cluster cases underscore the importance of targeted surveillance and intervention strategies while the wide range of R_0_ values observed in large outbreaks points to inconsistent control measures and emphasizes the need for strengthened vaccination campaigns and improved outbreak response protocols to better contain measles spread.

## 1. Introduction

Measles is a highly contagious viral disease that, despite being preventable through vaccination, continues to cause outbreaks around the world, with Europe noting March 2025 as having the highest number of cases reported in over 25 years [1]. One of the most important epidemiological phenomena associated with measles is the occurrence of clusters, groups of related cases that emerge within a defined geographic area or population and evolve over a specific period. These clusters provide a wide range of important insights such as transmission dynamics, public health-related vulnerabilities, and the effectiveness of immunization strategies.

Clusters can range from a few cases within a single household (typically at least three cases) to dozens or even hundreds within a community, school, or urban district. Smaller clusters may sometimes serve as the initial signal of a larger outbreak and warrant immediate public health investigation, with a single measles case being already reason enough for a prompt alert in Romania [2].

Distinctive features of measles clusters include [3]

(a) spatial proximity: cases frequently exhibit geographical clustering, commonly appearing within specific neighborhoods, educational institutions, or workplaces;
(b) temporal link: symptoms typically begin within a specific timeframe, generally ranging from days to weeks, and
(c) an epidemiological connection: individuals who form clusters frequently share social, familial, or occupational connections, which can enhance the probability of either direct or indirect transmission.

Several factors contribute to the emergence of measles clusters and the most significant is low vaccination coverage within a population [4]. Measles requires a high level of immunity—typically at least 95%—to achieve “herd immunity,” which prevents sustained transmission. When vaccination rates fall below this threshold, clusters can form and grow rapidly. As Romania experiences a loss in vaccination coverage, with the first MMR dose having a coverage below 95% since 2010 [5], the probability of clusters formation during a measles outbreak increases significantly.

The spread of measles within clusters is characterized by rapid transmission and a tendency for secondary cases to occur among susceptible contacts. The incubation period for measles is typically 10–14 days (but can vary from 6 to 21 days), and infected individuals are the most contagious from four days before to four days after the appearance of the characteristic rash.

Measles exhibit a high basic reproduction number R_0_ [6] and, as such, can create a rapidly growing cluster that, in the absence of prompt and effective control measures, can expand from being confined to a family or a school to a full-blown community-wide outbreak [7,8]. From this perspective, measles clusters represent both a warning sign and an opportunity for public health intervention. Left unchecked, clusters can and will escalate into widespread outbreaks, placing unvaccinated individuals, infants too young for vaccination, and immunocompromised persons at serious risk. The consequences include increased hospitalizations, complications such as pneumonia and encephalitis and, in severe cases, death.

Clusters also exhibit long-reaching consequences beyond the infections themselves by straining healthcare resources, disrupting education and community activities, and eroding an already negatively affected public confidence in health systems.

Early identification of clusters relies on vigilant local surveillance systems with healthcare providers being urged to promptly report suspected cases. Case and cluster investigations involve confirming diagnoses with laboratory testing (testing the first 5–10 cases is crucial, additional cases being confirmed based on clinical case definition and an epidemiological link to a confirmed case [9]), mapping epidemiological links between cases, assessing vaccination histories and identifying susceptible contacts for targeted intervention.

Due to heterogeneity of how measles spreads, assessing the local specificity of measles clustering may aid in improving how public health decisions are approached. The basic reproduction number for measles transmission is commonly estimated to be between 9 and 18 [6]. Calculating this figure for large outbreaks in Romania may help assess the effectiveness of surveillance and control measures and provide information on potential areas for improvement. By comparing cluster cases with non-cluster cases, gaps in immunization can be further highlighted, along with specifics that may lead to clustering.

## 2. Materials and Methods

The National Centre for Communicable Diseases Surveillance and Control manages the national measles database, supported by regional centers under the Romanian National Institute for Public Health. Public Health Directorates collect data from medical practitioners who suspect measles based on clinical signs. The collected forms include demographic and disease details, complying with GDPR. Potentially sensitive data, such as cluster names, which may include last names (for familial clusters), hospital names or district/town names, have been replaced with generic formulations, such as ‘cluster 1–county’.

**Figure 1:**
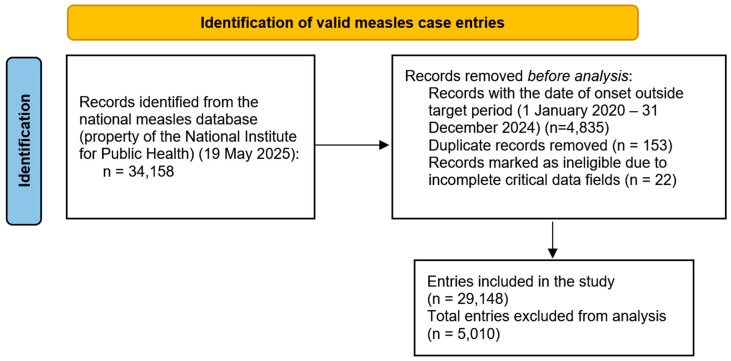
PRISMA diagram of case selection process.

A total of 29,148 entries in the national measles dataset have been considered for a retrospective study (Figure 1), which met the following criteria: date of onset between 1 January 2020–31 December 2024 and had all the relevant fields filled in the submission form. The data used has been provided by the National Institute for Public Health of Romania, and the study has been approved by the University ‘Dunărea de Jos’ Galați’s ethics committee.

The provided data was manipulated using Python v3.13, an increasingly popular tool in large-volume data management in healthcare and biology [10]. Cases were separated into outbreak and non-outbreak cases (with cluster-related cases being defined as part of clusters with at least 3 related cases based on the cluster status and case counted based on the field containing the name of the cluster filled).

The descriptive analyses have been performed using seaborn v0.13.2. The clusters consisting of 150 or more cases have been extracted and analyzed individually to provide deeper insights into how they emerged and evolved over time. Since estimates of the basic reproduction number (R_0_) were central to the analysis, a threshold of 150 cases in a single cluster was selected to reduce potential biases. This approach helps minimize the impact of factors that may artificially lower R_0_ values, such as local depletion of susceptible individuals or the presence of immunized groups, which could otherwise result in distinct smaller clusters that are actually interconnected but appear separate due to temporarily reduced R_0_ estimates. For instance, a household cluster with 10 cases can result in 1–2 cases in another household where most individuals are immunized; these 1–2 cases may then form a subsequent cluster of 12 cases in a different household. Although these clusters of 10 and 12 cases are often described separately, they collectively represent a single cluster comprising 23–24 cases. Addressing large-scale outbreaks that have spread throughout the community increases the likelihood of mitigating this problem.

To calculate the basic reproduction number, the formula used was R_0_ = exp(r × T), where r is the early exponential growth and T is the generation interval in weeks. The early exponential growth was calculated by first selecting the initial three weeks of each outbreak with higher-than-zero reported cases. For each week, the number of cases was transformed using the natural logarithm to linearize the exponential growth pattern. A linear regression was then performed with the week index as the independent variable and the log-transformed case counting as the dependent variable. The slope of this regression line represents the weekly exponential growth rate.

The maximum likelihood estimation (MLE) approach has been considered for calculating the basic reproduction number, as it makes use of the complete incidence data and can provide results that are statistically robust and accurate. However, as outbreaks evolved over long periods with time intervals in which only a few cases were reported, the MLE proved unreliable with estimates closer to 1, pointing to data incompleteness of the observed clusters (after the initial phase, the clusters may have created pockets of smaller clusters disconnected from the initial large one. Conversely, measures taken for limiting the outbreak could have proven effective, limiting the spread after the initial unconstrained phase). The early exponential growth method was preferred as the plotted data clearly showed an exponential trend.

## 3. Results

A total of 7011 cases (24.05%) were part of a cluster. The majority of cases were reported in community settings (4628 cases), followed by family environments (1986 cases), school-related settings (171 cases), hospitals (157 cases), childcare facilities (49 cases), and military groups (6 cases).

**Figure 2:**
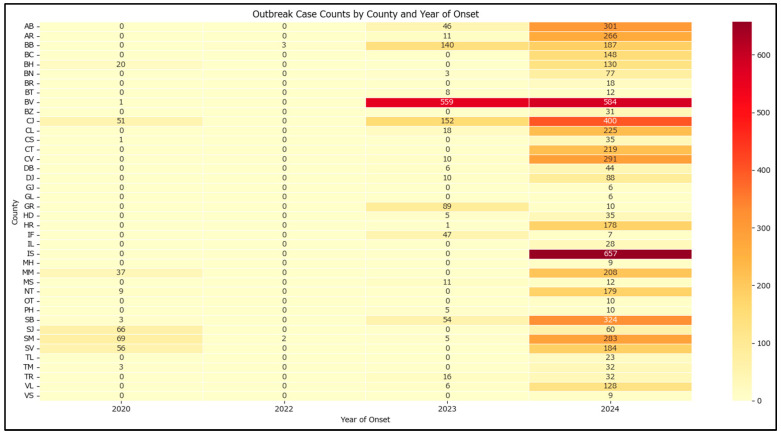
Heatmap of outbreak case counts by county and year of onset in Romania in 2020–2024.

There are significant disparities in how cluster-related cases are distributed across the country, with the county and year-based analysis (Figure 2) showing that Brașov is the most affected county, followed by Iași and Cluj with a high heterogeneity among areas affected.

Outbreak cases are associated with a higher chance of hospitalization and are more common in rural areas. The hospitalization rate is higher for outbreak cases (about 84%) compared to non-outbreak cases (about 74%) and the proportion of urban cases is lower in outbreaks (38%) than in non-outbreaks (48%). Both differences are statistically significant, with *p*-values lower than 0.001 (Table 1).

**Figure 3:**
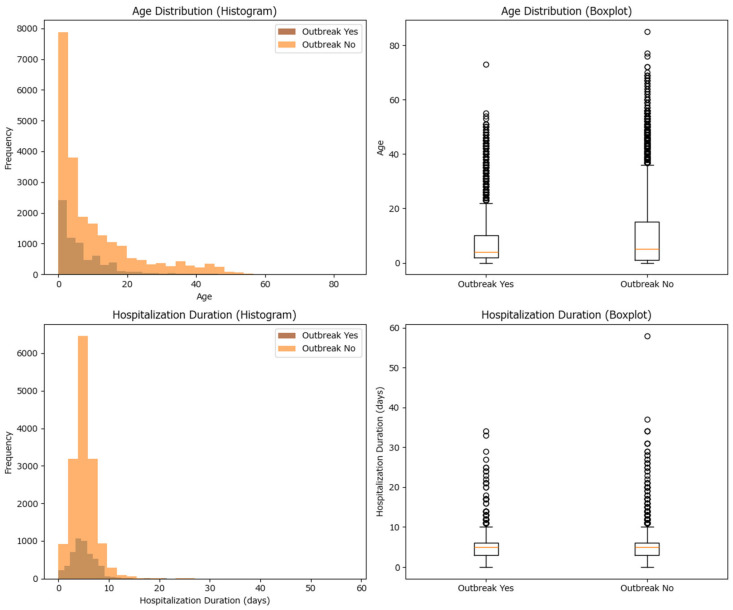
Histogram and box-plot of the case distribution among cluster and non-cluster cases based on age and hospitalization duration for measles cases in Romania in 2020–2024.

The median and average (mean) values for age and hospitalization duration have been calculated for both outbreak and non-outbreak cases. For age, the median is 4 years in outbreak cases and 5 years in non-outbreak cases, with means of approximately 7.13 and 10.09 years, respectively. For hospitalization duration, the median is 5 days in both groups, with means of approximately 4.94 days (outbreak) and 4.76 days (non-outbreak). Statistical testing using the Mann–Whitney U test shows that the differences in age and hospitalization duration between the two groups are statistically significant (*p*-values much less than 0.05), which indicates that outbreak and non-outbreak cases differ in their age and hospitalization duration distributions (Table 2 and Figure 3). While both hospitalization duration and age differ significantly between outbreak and non-outbreak cases, the magnitude of these differences is very small, suggesting limited practical impact.

**Figure 4:**
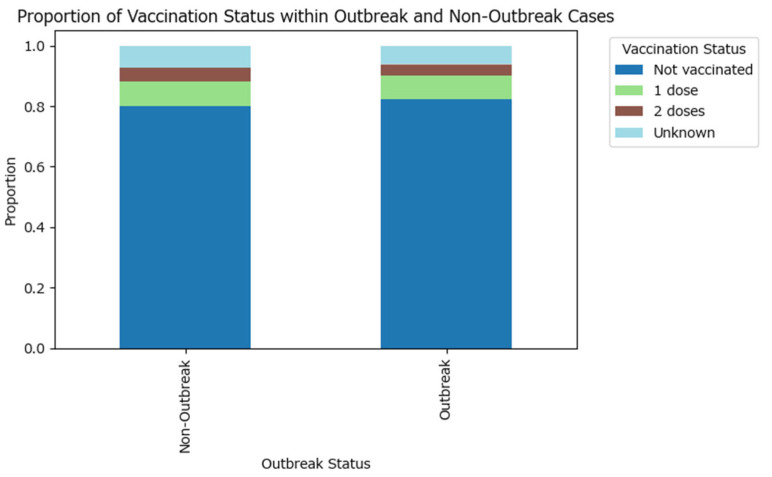
Proportion of vaccination status within measles outbreak and non-outbreak cases in Romania in 2020–2024.

A higher proportion of outbreak cases are not vaccinated compared to non-outbreak cases, while non-outbreak cases have relatively higher proportions of individuals with at least one dose of the vaccine (Figure 4). A similar proportion of outbreak-related cases have an unknown vaccination status (6.12%) compared to the non-outbreak cases (7.12%).

The visual findings are supported by the Chi-squared test (test statistic is approximately 30.74 with 4 degrees of freedom, and the *p*-value is <0.0001, which is much less than the conventional significance threshold of 0.05), which confirms that the difference in vaccination status distributions between the two groups is statistically significant, although the effect size is weak (Cramér’s V test value of 0.032). These results suggest that vaccination status is partly associated with outbreak occurrence, emphasizing the importance of vaccination in preventing outbreaks.

**Figure 5:**
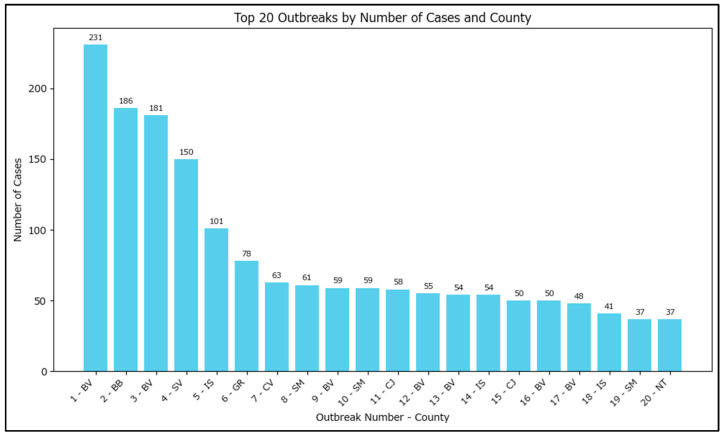
Top 20 outbreaks by number of cases in Romania in 2020–2024. Note: the outbreak names had been redacted to ‘<current number–based on the outbreak size>–<county of incubation>’ with the actual name of the outbreaks potentially allowing for specific identification of the locations/families involved.

The number of cases within an outbreak varies greatly from 3 to 231. The top 20 highest case counts outbreaks have been plotted, with some outlier outbreaks having a very large number of measles cases (Figure 5).

### Large Outbreaks Analysis

Outbreaks with 150 or more cases have been identified and analyzed. Across these outbreaks, there are 750 total cases, with most occurring in urban environments. The affected population is predominantly young, as indicated by low mean and median ages (Table 3).

**Figure 6:**
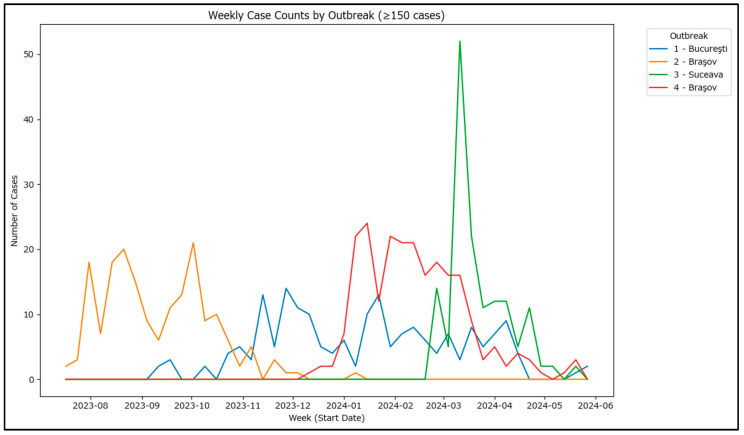
Weekly case counts in 4 largest outbreaks in Romania in 2020–2024.

Weekly aggregation of cases shows distinct peaks for each outbreak, highlighting periods of rapid case increase and potential differences in how each outbreak was approached (Figure 6). To assess the differences, these outbreaks allow for an estimation of the basic reproduction number as the cases are affiliated with each other. The basic reproduction number (R_0_), which requires information on the generation time or serial interval (i.e., the average time between successive cases in a transmission chain) and an appropriate estimation method (e.g., exponential growth method, maximum likelihood estimation or Bayesian inference), has been calculated for the four outbreaks.

For the estimation method, the exponential growth method has been chosen, and early exponential growth phase has been calculated for each of the four outbreaks by selecting the initial consecutive weeks with increasing case counts for each outbreak. After the period identification for each outbreak, the weekly case counts have been log-transformed, and a linear regression model has been fitted to estimate the exponential growth rate (r) for each outbreak (Table 4).

For generation time, a range of 5 days [9:13 days] has been selected as measles can show high variability in the incubation period, especially among the unvaccinated [11]. As the exponential growth rate has been calculated based on the weekly cases, the generation time has been converted from days to weeks (i.e., a GI of 9 days becomes 9/7 ≈ 1.29 weeks and, similarly, 13 days become 13/7 ≈ 1.86 weeks).

For each outbreak and for each GI value in the range [9, 10, 11, 12, 13] days, R_0_ was calculated using the formula R_0_ = exp(r × T), where T is the generation interval in weeks. This gives a range of R_0_ values for each outbreak, reflecting the uncertainty in the generation interval using confidence intervals (Table 5).

**Figure 7:**
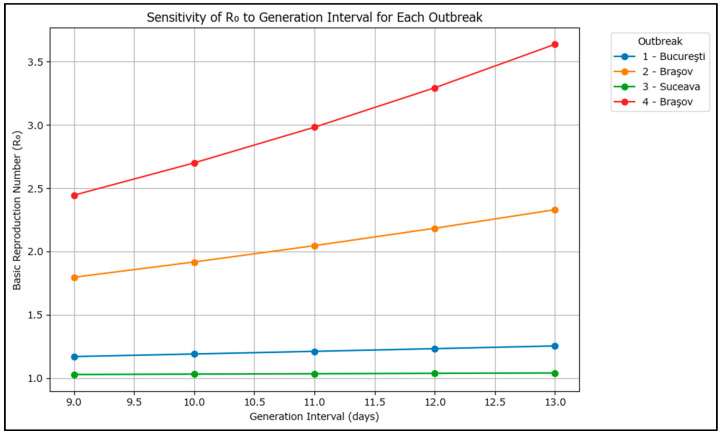
Line plot of R_0_ versus generation interval for each large measles outbreak in Romania in 2020–2024.

For easier interpretation, the results have also been plotted graphically in Figure 7.

For each outbreak, R_0_ increases as the assumed generation interval increases, which suggests that the circulating measles genotype induces a longer incubation period, which puts further pressure on the surveillance system.

The results show that outbreaks 2 and 4 (Braşov) had higher transmission potential, while Suceava’s outbreak had the lowest. The findings are consistent with the limited control exerted in the Brașov area as the outbreaks occurred in high-risk Roma communities and an R_0_ of 3.5 may more closely resemble the overall situation in Romania’s current outbreak.

The two Brașov outbreaks also showed higher mean disease duration (5.50 and 5.18) when compared to București (4.65) or Suceava (4.70), and also had higher mean hospitalization duration (5.65 and 5.44) compared to București (4.67) or Suceava (3.66).

Vaccination, although limited in these outbreaks (7.6% of the cases vaccinated with at least one MMR dose), did provide a lower disease duration and hospitalization (Table 6). Albeit with a much lower case count, the protective effect was less visible in urban areas with outcomes similar to those unvaccinated from the rural area, which raises concerns about the data accuracy of the immunizations being performed.

## 4. Discussion

The recent years, marked by recurrent measles outbreaks, have tested Romania’s immunization strategies and healthcare infrastructure, continuing to be a challenge to the country’s public health system. Between 2020 and 2024, Romania experienced a series of measles outbreaks of varying scale and duration, revealing critical gaps in vaccination coverage, especially among vulnerable populations. By examining the underlying factors contributing to outbreak persistence and spread such as epidemiological trends, vaccine uptake and clinical outcomes, we gain a deeper understanding of the ongoing challenges and potential solutions for measles prevention and control in Romania.

One of the most significant findings in this research is the persistent occurrence of large clusters, particularly in urban settings such as Bucharest, where outbreaks lasted up to several months, a duration likely attributable to the dense population and pockets of low MMR vaccine coverage [12], while others occur in rural areas within marginalized Roma communities. These findings are consistent with earlier studies [13], underlining the challenges faced in initiating and sustaining public health interventions in such populations, where the involvement of mediators and culturally tailored approaches are essential.

School environments may have played a substantial role in the genesis of the several large outbreaks identified in the current study, as suggested by the lower median age among cluster-related cases and the timing of outbreaks shortly after school reopening. This aligns with previous research [14], indicating that school-related clusters can involve a greater number of children. However, it is notable that the outbreak sizes in our study were generally smaller than those reported elsewhere [15], possibly reflecting differences in population immunity or intervention strategies.

Hospital-associated outbreaks were less frequently observed in our dataset, but the risk of nosocomial transmission remains a concern [16], particularly given the likelihood of underreporting due to the challenges in identifying exposures during the prodromal phase. In contrast, a significant proportion of cases were family-related, with rural areas predominantly affected, suggesting that household and community transmission pathways remain vital in sustaining outbreaks. Earlier simulations have demonstrated that household measles transmission diminishes over time in conjunction with decreasing household sizes, a pattern linked to reduced fertility rates [17].

The basic reproduction number (R_0_) exhibited considerable variability in our measles clusters, with values remaining high even among clusters with some vaccinated individuals and requiring assessment within the context of local conditions [6], similar to findings from other researchers [18]. This indicates that while control measures were implemented, their effectiveness was inconsistent, highlighting gaps in outbreak response and the continued vulnerability of non-immunized populations. The sustained high R_0_ in some clusters suggests that targeted interventions may not have reached all at-risk groups or that compliance with control measures was suboptimal.

For efficient measles cluster control, a series of measures, such as the ring vaccination, targeted immunization of contacts and at-risk groups to contain transmission, as well as efforts to quarantine within high attack rates have been met with a mixed reception in Romania, especially in the high-risk areas already suffering from low vaccine coverage, such as Roma communities. Addressing them requires culturally competent communication and collaboration with community leaders to build trust and promote vaccination. Outbreak communication and providing clear, timely information to affected communities to support compliance and reduce panic is one area which can be improved upon substantially with minimal resource investment. Enhancing measles surveillance by intensifying monitoring for new cases based on forecasting data is also a strategy that has not been previously explored.

Several obstacles to effective outbreak management were identified, including vaccine misinformation, logistical challenges, and disparities in healthcare access, especially in rural areas. The lack of general practitioners in many rural communities [19] and the resulting inequities in healthcare services likely contribute to the higher prevalence of outbreaks [20] and the difficulty in achieving adequate vaccine coverage, with primary healthcare playing a critical role [21]. Furthermore, the finding that a similar proportion of cases in both outbreak and non-outbreak settings had unknown vaccination status points may point to systemic issues in data collection and record-keeping, rather than a lack of interest from healthcare providers in providing timely, complete datasets for measles patients.

The one-dose MMR vaccine approach, as simulated by our data with a large number of measles cases occurring among children that were not yet eligible for the second MMR dose, proves insufficient in preventing outbreaks and reinforcing the need for comprehensive immunization strategies that address not only public health policy but also educational and socio-economic factors. In particular, tertiary education [22] and occupational mobility [23] were identified in other studies as influencing complete immunization rates among children, suggesting that efforts to increase vaccine uptake must also consider broader social determinants of health.

Looking forward, whole-genome sequencing may prove useful in the context of clustering as it provides insight into how the outbreak evolved by providing cluster-defining mutations [24] and is currently a perspective already being explored in Romania [25]. Cross-referencing the measles database with the national electronic register of vaccinations as a prospective possibility may enhance outbreak detection and response, enabling faster identification of high-risk institutions and more effective containment measures.

Finally, the phenomenon of falsified immunization data, first described in the context of COVID-19 vaccination campaigns [26], raises concerns about the reliability of some of the identified vaccination records, particularly in urban settings where worse-than-expected outcomes were observed among the few vaccinated cases. Assessing and eventually addressing this issue requires robust verification mechanisms and continued vigilance by public health authorities.

### Limitations

While the dataset does not contain missing critical data points, being internally maintained, data availability may be acting as a limiting factor in several ways:

(a) Patients may not be aware of or are unwilling to share information regarding a potential infective contact, thus limiting classifying the case as being cluster-related. This also depends on medical staff’s ability to build trust with patients, tailor communication to their social and ethnic backgrounds, and collect timely data while the patient remembers key details.
(b) There is limited data collected throughout a cluster’s evolution. Most of the clusters exhibit a specific behavior also highlighted in the large cluster analyses, which is an exponential increase in case counts in the initial timeframe with a lower sustained transmission with R_0_ closer to 1 until the cluster’s closure. No data is available to describe why this phenomenon occurs, with multiple potential explanations: the cluster may be naturally diminishing by exhausting susceptible individuals, the measures taken limiting the spread of the measles may be effective to a certain extent or, as the cluster starts halting, cases may be escaping from the original cluster and instead become grouped in smaller and apparently unrelated clusters. These are fundamentally different ways of how a cluster may evolve and have a profound effect on the basic reproduction number estimation.
(c) Other local specificities such as socio-economic status, healthcare literacy, healthcare and vaccine access, population density and recent travels are factors contributing significantly to how an airborne infectious disease spreads and which can be accounted for tangentially in our analysis.

## 5. Conclusions

Measles outbreak cases from 2020 to 2024 in Romania are associated with a higher chance of hospitalization and are more common in rural areas. Large outbreaks are characterized by a high burden among young, unvaccinated, urban populations, with moderate disease severity and hospitalization. These findings can inform targeted interventions and resource allocation for outbreak control.

## Figures and Tables

**Table 1:** Hospitalization rate and living environment of the outbreak and non-outbreak cases in Romania in 2020–2024.

	Outbreak	Non-Outbreak	Cramer’s V	*p*-Value
Hospitalization rate	83.68%	74.46%	0.091	<0.001
Urban setting	37.62%	48.39%	0.092	<0.001

**Table 2:** Median, mean, and statistical significance for age and hospitalization duration for measles cases in Romania in 2020–2024.

	Outbreak Median	Outbreak Mean	Non-Outbreak Median	Non-Outbreak Mean	Rank–Biserial Correlation	Mann–Whitney U *p*-Value
Age	4	7.1	5	10	0.044	<0.001
Hospitalization duration	5	4.9	5	4.7	−0.041	<0.001

**Table 3:** Spatial and demographic breakdown for large outbreaks in Romania in 2020–2024.

No.	Name of the Outbreak	Macroregion of Notification	Living Environment	Case Count	Mean Age	Median Age
0	1—BB	Bucuresti-Ilfov	URBAN	185	8.2	4
1	1—BB	Sud-Muntenia	URBAN	1	15	15
2	2—BV	Centru	RURAL	179	6.2	5
3	2—BV	Centru	URBAN	2	2.5	2.5
4	3—SV	Nord-Est	RURAL	150	9.3	8
5	4—BV	Centru	RURAL	9	4.3	2
6	4—BV	Centru	URBAN	222	3.9	2

**Table 4:** Exponential growth rate estimation for each large measles outbreak in Romania in 2020–2024.

No.	Outbreak	Exponential Growth Rate (r)	Std Error	R^2^
0	1—Bucureşti	0.121	0.076	0.384
1	2—Braşov	0.455	0.142	0.718
2	3—Suceava	0.020	0.208	0.002
3	4—Braşov	0.695	0.090	0.936

**Table 5:** R_0_ estimates with 95% confidence intervals for each large measles outbreak in Romania in 2020–2024 and generation interval [9:13 days].

Outbreak	Generation Interval (Days)	Estimated R_0_	R_0_ Lower 95% CI	R_0_ Upper 95% CI
1—Bucureşti	9	1.169	0.963	1.419
1—Bucureşti	10	1.189	0.959	1.475
1—Bucureşti	11	1.210	0.955	1.533
1—Bucureşti	12	1.231	0.951	1.594
1—Bucureşti	13	1.253	0.947	1.658
2—Braşov	9	1.796	1.253	2.573
2—Braşov	10	1.917	1.285	2.858
2—Braşov	11	2.045	1.318	3.174
2—Braşov	12	2.183	1.351	3.526
2—Braşov	13	2.330	1.386	3.916
3—Suceava	9	1.027	0.607	1.737
3—Suceava	10	1.030	0.574	1.847
3—Suceava	11	1.033	0.543	1.963
3—Suceava	12	1.036	0.514	2.088
3—Suceava	13	1.039	0.486	2.220
4—Braşov	9	2.444	1.944	3.073
4—Braşov	10	2.700	2.094	3.481
4—Braşov	11	2.982	2.254	3.944
4—Braşov	12	3.293	2.427	4.468
4—Braşov	13	3.637	2.614	5.061

**Table 6:** Grouped summary by vaccination and environment for large outbreaks in Romania in 2020–2024.

No.	Is Vaccinated	Is Urban	Mean Disease Duration (Days)	Mean Hospitalization Duration (Days)	Case Count
0	FALSE	FALSE	5.2	5.2	298
1	FALSE	TRUE	4.9	5.0	395
2	TRUE	FALSE	4.6	3.4	41
3	TRUE	TRUE	5.5	6.6	16

## Data Availability

The original contributions presented in this study are included in the article. Further inquiries can be directed to the corresponding author(s).
